# Associations between mortality and meteorological and pollutant variables during the cool season in two Asian cities with sub-tropical climates: Hong Kong and Taipei

**DOI:** 10.1186/1476-069X-12-59

**Published:** 2013-07-19

**Authors:** William B Goggins, Emily YY Chan, Chunyuh Yang, Marc Chong

**Affiliations:** 1 Division of Biostatistics, School of Public Health and Primary Care, Chinese University of Hong Kong, Shatin, Hong Kong; 2 Division of Family Medicine, School of Public Health and Primary Care, Chinese University of Hong Kong, Shatin, Hong Kong; 3 Institute of Public Health, Kaohsiung Medical University, Kaohsiung, Taiwan

**Keywords:** Cold wave, Cold spell, Biometeorology, Poisson regression, Mortality

## Abstract

**Background:**

Numerous studies have found associations between extreme temperatures and human mortality but relatively few studies have been done in sub-tropical and tropical cities, especially in Asia. In this study we examine the impact of cold temperatures, cold waves and other meteorological and environmental variables on cool season mortality in 2 subtropical Asian cities.

**Methods:**

Separate analysis of daily mortality time-series from Hong Kong and Taipei using Generalized Additive Models with natural mortality as the outcome daily mean temperature as the main explanatory variable and relative humidity, solar radiation, wind speed, pollutants (nitrogen dioxide (NO_2_), sulfur dioxide (SO_2_), respirable suspended particulates (PM_10_), ozone (O_3_), seasonality and day of the week controlled as potential confounders. Lags up to 35 days were considered for temperature, and distributed lag models were used to determine the number of lags for final models. Subgroup analyses were also done by gender, age group, cause of death and geographical area of residence.

**Results:**

Cold temperatures were strongly associated with higher mortality with lagged effects persisting up to 3 weeks in Hong Kong and 2 weeks in Taipei. Cold effects were much stronger for deaths among older people and non-cancer deaths. Prolonged cold spells modestly but significantly raised mortality after accounting for the effects of individual cold days. Higher daily ozone levels were also strongly associated with higher short-term mortality in Taipei and Hong Kong, while relative humidity and solar radiation were weakly and inconsistently associated with mortality.

**Conclusions:**

Cold temperatures and cold spells substantially increase short-term mortality in sub-tropical Asian cities particularly among the elderly. Greater attention needs to be paid to the adverse health effects of cold temperatures. Interventions including provisions of shelters, cold weather warnings and education about the possible health effects of cold temperature should be carried out in sub-tropical areas.

## Background

Global climate change has led to an increased interest in the effect of weather and climate on human health with the health effects of heat and heat waves being a particular focus. However the effects of cold weather on health are also important and should not be forgotten. A short-term association between temperature and mortality from natural causes has been noted in many previous studies with mortality increases generally being noted for both hot and cold temperatures. However the actual thresholds and magnitude of heat and cold effects have been found to vary between locations, with heat effects being generally stronger in colder climates and cold effects stronger in warmer climates [1-4]. Specific studies of cold temperatures effects on mortality include a recent multi-city European study [2] which found stronger cold effects for older people, and for those living in warmer climates, and that cold temperature effects persisted at lags up to 23 days [2]. A multi-city study of temperature effects in cities in Thailand, India, Latin America and Eastern Europe [4] found that heat and cold effects on mortality were apparent in almost all of the cities considered but that the threshold at which these effects became apparent varied considerably. A recent study of associations between apparent temperature and mortality in Beijing, Taipei, Seoul and Tokyo [5], found strong heat effects on mortality with thresholds ranging from 30-31°C. However they did not examine cold effects in detail and did not consider lags beyond 2 days, a serious limitation for studies of cold effects on mortality. A study conducted using data from rural areas of Bangledesh estimated a 3.2% increase in all-cause mortality for each 1°C. drop (average of lags 0–13 mean temperature) below a threshold of 21°C [6].

In addition to modeling the temperature-mortality association some studies have looked at measuring the specific effect of cold spells, defined as sustained periods of cold temperature over a period of several days (the number of days and thresholds vary between studies). A study using data from the Netherlands [7] estimated a 12.8% increase in natural mortality during cold spells, and found greater excess for mortality due to cardiovascular causes and mortality in those > 65 years of age. In contrast, a Czech study [8] found excess mortality during cold spells of about 6.3% overall with the greatest excess for men aged 25–59. A study done in Moscow reported significant increases in mortality of 8.9% and 9.9% for those in the 75+ age group during two recent cold spells [9]. There was no significant increase for those under 75 years old observed in this study. A recent study from China which examined the effects of a prolonged cold spell in 2008 on mortality in three cities in Guangdong province, a southern Chinese province adjacent to Hong Kong, estimated a 60% increase in mortality due to the cold spell in two of three cities examined with larger increases for females, the elderly and for respiratory mortality [10].

Hong Kong and Taipei have subtropical climates and generally experiences mean daily temperatures varying between 10–20 degrees °C during the December-March cool season. While cold temperatures usually only last for a few days, in 2008 Hong Kong experienced it’s longest cold spell in 40 years, with mean temperatures < 14 degrees °C for 24 consecutive days. Compared to Hong Kong, Taipei has slightly more temperature variation and considerably more rainfall during the cool season. While Taiwan has a lower per-capita income than Hong Kong, $US35,700 vs. $US45,900 (2010 purchasing power parity estimates) [11], both are considered high income countries, with levels of economic development comparable to that of the European and North American countries in which most similar studies have been conducted. Hong Kong and Taiwan have roughly similar distributions of causes of death with malignant neoplasms being the number one cause of death in both places and heart disease second [12,13]. The health care systems in both study locales have a strong public element. The Hong Kong Hospital Authority, a public organization, accounts for more than 90% of hospital admissions [14] and their fees are heavily subsidized. Taiwan implemented the compulsory National Health Insurance (NHI) in 1995, which extended coverage to all residents [15].

While an association between cold temperatures and higher mortality is well established, relatively few studies have examined the independent effect of cold waves, after controlling for the effect of individual cold days. In addition few studies have examined the association between mortality and other meteorological variables such as relative humidity, solar radiation and wind speed. In this study we use modern statistical methods to investigate the associations between mortality and temperature, cold waves, and other meteorological and pollutant variables during the cool season in Hong Kong and Taipei.

## Methods

### Data

For Hong Kong mortality, meteorological and pollutant data were obtained from the Hong Kong Census and Statistics Bureau, Hong Kong Observatory and Hong Kong Environmental Protection Department, respectively. For Taipei mortality data was obtained from Taiwan’s Department of Health, the weather data from Taiwan’s Central Weather Bureau and the pollutant data from the Taiwanese Department of Environmental Protection Administation. Meteorological variables collected from both locations included mean daily temperature (°C), mean daily relative humidity (%), mean daily wind speed (km/hr) and total global solar radiation (MJ/m^2^). Pollutant variables included respirable suspended particles with diameter ≤ 10 μm (PM_10_), nitrogen dioxide (NO_2_), ozone (O_3_), and sulfur dioxide (SO_2_). PM_10_ was measured in μg/m^3^ while the three gaseous pollutants were measured as μg/m^3^ in Hong Kong and parts per billion (ppb) in Taipei.

Ethics approval for this research was obtained from the Joint Chinese University of Hong Kong- New Territories East Cluster Clinical Research Ethics Committee (2011.337).

### Statistical analysis

Descriptive statistics were calculated for all important variables including deaths, overall and by gender, age group and cause. Poisson Generalized Additive Models (GAMs) for time-series [14] were used to model the independent associations of non-accidental mortality (ICD-9 codes 001–799; ICD-10 codes: A00-T99, Z00-Z99) with the meteorological and pollutant variables while controlling for confounders. The analyses covered the years 1999–2009 for Hong Kong and 1999–2008 for Taipei. Analyses were restricted to the cool season (November-March). Initially distributed lag models [16] were used to examine the lag structure of the temperature effects with lags up to 35 days considered. The initial distributed lag terms used smooth terms with maximum 5 degrees of freedom (df) to model the association between the meteorological variables and pollutant variables and mortality, while the lagged effects for these variables were polynomial (4 df) constrained, Other meteorological variables were then modeled using smooth terms with the averages of the same lags as those used as for temperature. Seasonality was adjusted by creating a ‘day of the year’ term, DOY = 1 for January 1, …., = 365 (or 366) for December 31. The DOY term was allowed a maximum of 4 degrees of freedom while day of the week (DOW) was adjusted for using indicator variables. Long term trends were adjusted for by including a term, t = 1 (January 1, 1999), …, 4018 (December 31, 2009, Hong Kong) or 3625 (December 31, 2008 for Taipei), with maximum 4 degrees of freedom.

The specific effect of cold waves was modeled using a separate indicator variable for the period of the cold wave. The indicator variable was = 1 for the days during which the cold wave occurred, starting with the 5^th^ day, plus a lag period of 20 days after the end of the cold wave. Thus for instance for the 2008 Hong Kong cold wave which lasted from January 25^th^ through February 16^th^, the cold wave indicator = 1 for consecutive days from January 29^th^ through March 8^th^. Cold waves were defined as periods with at least 5 consecutive days with mean temperature below the cool season 10^th^ percentile of mean temperatures, which was 14.1°C. for Hong Kong and 13.8°C. for Taipei.

Separate models fit for demographic subgroups, including subgroups defined by age (45–64, 65–74, 75–84 and 85+), and gender, were used to determine if the effect of cold temperature and cold waves was stronger for some population groups than for others. We also fit separate models for specific common causes of death in Hong Kong including cancer, cardiovascular diseases, pneumonia, stroke, diabetes, nephritis/nephrosis and chronic lower respiratory diseases.

The R statistical software was used for the analysis with the contributed package MGCV [17] used to fit the GAMs. The MGCV package uses generalized cross-validation to select the degrees of freedom for each smooth (non-linear) term and thus the user only needs to provide the maximum degrees of freedom for each variable [17]. The initial distributed lag models were fit using MGCV and DLNM [16].

### Sensitivity analyses

We also performed several sensitivity analyses. While no information on influenza was available for Taipei, weekly influenza consultation rates per 1000 consultations among general practitioners were available for Hong Kong. This variable was not included in the main models for Hong Kong as we wanted to make the methodology used for the two cities as comparable as possible, However we did a sensitivity analysis to see if the inclusion of this variable impacted other model estimates. We also did sensitivity analyses to examine whether varying the maximum df for the time trend and season terms in the models affected other parameters and whether the use of more flexible modeling of temperature effects had a substantial impact on the cold wave parameters.

## Results

Descriptive statistics for the study variables for both cities are shown in Table 1. The median number of daily deaths during the cool season was 91 and 33 in Hong Kong and Taipei, respectively. In both cities male deaths outnumbered female deaths and the age group with the largest number of deaths was 75–84 years of age. The median and IQR for mean temperatures were nearly identical: Hong Kong (18.9°C, 16.5-20.9°C) and Taipei (18.8°C, 16.4-21.1°C). Relative humidity levels were also similar while total solar radiation and mean wind speed were considerably higher in Hong Kong. PM_10_ levels were slightly higher in Hong Kong than Taipei. Comparison between the other pollutants is hampered by the use of different units but NO_2_ levels appear to have been roughly similar, while Hong Kong had higher SO_2_ levels, and lower O_3_ levels than Taipei.

**Table 1:** Descriptive statistics for the study cities, cool season (Jan-March and Nov-Dec) from 1999–2009 for Hong Kong and 1999–2008 for Taipei

	**Hong Kong**			**Taipei**		
**Variable**	**Median**	**25**^**th**^**% ile-75**^**th**^**% ile**	**5**^**th**^**%ile-95**^**th**^**% ile**	**Median**	**25**^**th**^**% ile-75**^**th**^**% ile**	**5**^**th**^**%ile-95**^**th**^**% ile**
All Natural Deaths	91	82-101	71-118	33	28-37	23-45
Male deaths	51	45-57	37-67	21	17-24	13-29
Female deaths	40	35-46	28-56	14	11-17	8-21
Deaths 45-64	14	11-16	8-21	7	5-9	3-12
Deaths 65-74	19	16-23	12-28	7	6-9	3-13
Deaths 75-84	31	26-35	20-42	12	10-15	7-19
Deaths 85+	23	18-29	13-38	7	5-10	3-14
**Deaths by Cause**						
Cancer	31	27-35	22-41	11	9-13	6-17
Cardiovascular	16	13-19	9-25	9	7-11	4-15
Respiratory Infections	11	8-15	5-21	1	1-2	0-4
Chronic Respiratory	6	4-8	2-12	5	4-7	2-10
Stroke	6	4-9	2-13	4	2-5	1-7
Nephritis	3	2-5	1-7	1	1-2	0-4
Septicaemia	1	1-2	0-4	0	0-1	0-2
Diabetes	2	1-3	0-5	3	1-4	0-6
Cirrhosis	1	0-2	0-3	1	0-1	0-3
**Environmental Variables**						
Mean Temp °C	18.9	16.5-20.9	12.6-24.1	18.8	16.4-21.1	12.5-24.1
Mean Relative Humidity (%)	77.0	68-84	52-92	76.9	69-83	59-91
Total Global Solar Radiation	12.3	6.9-15.0	2.6-18.2	6.6	2.7-10.9	0.3-15.9
Mean Wind Speed Km/hour	24.3	17.9-30.4	9.3-38.9	10.1	6.5-13.3	3.6-17.3
O_3_ μg/m^3^ (HK) ppb (Taiwan)	37.0	26.1-48.9	13.3-65.8	21.8	17.1-27.1	10.6-34.9
NO_2_ μg/m^3^ (HK) ppb (Taiwan)	62.1	52.8-75.6	42.4-103.6	29	24.3-34.7	19.3-47.34
SO_2_ μg/m^3^ (HK) ppb (Taiwan)	16.6	12.2-22.8	7.9-37.3	3.6	2.6-5.1	1.5-7.7
PM_10_ μg/m^3^	62	46.4-80.2	29.9-111.4	49.5	34.5-70.2	22.7-105.7

### Hong Kong

**Figure 1:**
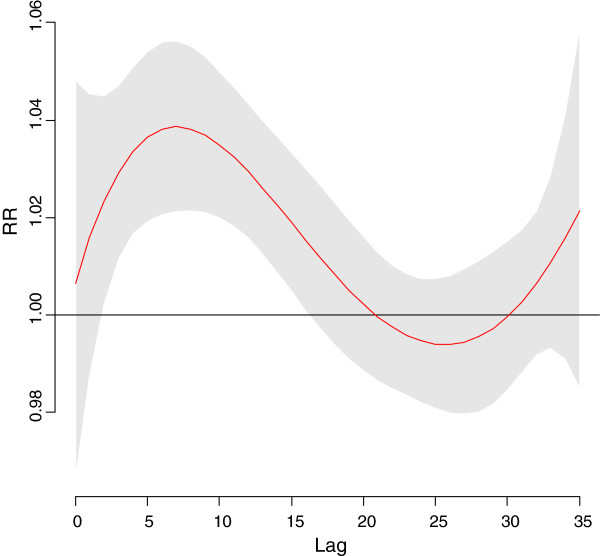
**Relative Risk for mortality by lag in Hong Kong for a 10°C. day vs, a 25°C. day.** LEGEND:. RR = Relative Risk. Relative Risk. 95% Confidence Region for Relative Risks.

The time course of the association between mortality and cold temperatures is shown in Figure 1. Based on the results of the distibuted lag model we then fit a models with a single smooth term representing the average of lags 0–20 mean temperature. Since the association of mortality with this term was approximately linear throughout the range of temperatures for the cool season, the final models used linear terms for average lag 0–20 mean temperature. The effect of cold temperatures peak about 7 days later but there is some increase out to about 20 days so the cumulative RR continues to increase up to that point. For pollutant variables distributed lag models indicated that the effect of O_3_ also lasted for about 20 days in Hong Kong so the average of lags 0–20 O_3_ was used in this model. Other pollutant variables were not significant predictors of mortality after adjustment for O_3_ and the meteorological variables so they were dropped from the final model after confirming they were not confounders. The results of the GAMs for mortality in Hong Kong are shown in Table 2. Lower average lags 0–20 mean temperatures, relative humidity and solar radiation and higher average lag 0–20 O_3_ levels were all significantly associated with higher all-cause mortality, with cold temperature effects being by far the strongest. The cumulative increase in mortality per 1°C decrease in mean temperature was 3.8%, which corresponded to a 45% increase per 10°C temperature decrease.

**Table 2:** Results of Poisson Generalized additive models without cold wave indicator: Hong Kong 1999–2009 cool season (January-March and November-December)

	**Lag 0–20 Mean Daily Temperature 10 C decrease**		**Lag 0–20 Mean Relative Humidity 10% decrease**		**Lag 0–20 Total Global Solar Radiation 10 W/m**^**2**^**decrease**		**Lag 0–20 O**_**3**_**10 μg/m**^**3**^**increase**	
	**% Increase (95%)**	**p**	**% Increase**	**p**	**% Increase**	**p**	**% Increase**	**p**
All	45 (39, 52)	<.001	2.0 (0.1, 3.8)	.035	12 (7, 18)	<.001	4.0 (2.8, 5.3)	<.001
Male	40 (33, 47)	<.001	1.9 (0.0, 3.9)	.057	13 (6, 21)	<.001	3.6 (2.1, 5.1)	<.001
Female	51 (42, 61)	<.001	1.5 (−1.1, 4.2)	.25	10 (3, 19)	.008	4.8 (3.1, 6.6)	<.001
Age								
45-64	17 (8, 27)	<.001	3.7 (0.3, 7.2)	.031	6 (−5, 19)	.30	1.6 (−0.3, 3.6)	.092
65-74	31 (20, 43)	<.001	6.3 (2.7, 10.0)	<.001	16 (5, 29)	.004	2.1 (−0.3, 4.6)	.079
75-84	47 (37, 56)	<.001	−0.3 (−3.7, 2.1)	.80	13 (4, 23)	.003	5.2 (3.3, 7.2)	<.001
85+	73 (61, 87)	<.001	0.7 (−2.4, 3.9)	.66	15 (5, 26)	.003	6.3 (4.1, 8.6)	<.001
Cause of Death								
Cancer	15 (6, 24)	<.001	3.0 (0.0, 6.0)	.040	8 (0, 18)	.065	0.8 (−0.6, 2.2)	.28
Cardiovascular Diseases^1^	56 (43, 70)	<.001	3.7 (0.0, 7.8)	.059	15 (3, 29)	.014	5.4 (3.5, 7.4)	<.001
Respiratory	73 (51, 98)	<.001	0.7 (−4.9, 6.5)	.82	20 (1, 43)	.036	7.7 (4.6, 10.9)	<.001
Respiratory infections	79 (60, 99)	<.001	−0.5 (−5.1, 4.4)	.84	8 (−6, 24)	.27	7.4 (5.0, 9.8)	<.001
Stroke	61 (36, 92)	<.001	−3.2 (−9.5, 3.5)	.34	−4 (−21, 16)	.65	−2.2 (−5.2, 0.9)	.17
Nephritis	53 (27, 84)	<.001	7.3 (−0.7, 15.9)	.072	14 (−11, 46)	.29	0.0 (−4.0, 4.0)	.99
Septicaemia	63 (25, 113)	.0011	4.4 (−7.0, 17.0)	.46	16 (−19, 65)	.42	0.3 (−5.3, 6.2)	.92
Diabetes	84 (42, 138)	<.001	0.0 (−9.0, 10.8)	.92	16 (−18, 63)	.41	2.6 (−3.3, 8.8)	.39
Cirrhosis	49 (9, 103)	.013	2.8 (−9.1, 16.2)	.66	5 (−32, 61)	.84	−3.6 (−10.3, 3.5)	.31

Stratified analyses by age at death showed clear trends for the magnitude of temperature and ozone effects to be greater for older age groups, while solar radiation was stronger for all age groups over 65 and RH% effects showed no consistent trend. Analyses stratified by cause of death showed that all common causes of non-cancer deaths were much more strongly affected by cold temperatures than cancer deaths. Cardio-respiratory deaths were more sensitive to higher ozone and lower solar radiation levels while no consistent patterns were noted for RH. Stratified analyses by gender showed little effect modification by this variable. The model additionally including an indicator for cold wave showed that the days within the cold wave period had an estimated average 4.0% (95% CI = 1.9%, 6.1%, p = .0001) mortality after adjustment for all other variables. Thus the estimated effect of a series of days with cold temperatures was significantly greater than the expected sum of effects of the individual days. The additional cold wave effect was also stronger for older age groups with average increases of 2.3%, 3.6%, 4.0% and 4.9% for the 45–64, 65–74, 75–84 and 85+ groups, respectively. The cold wave effect was weaker for the model with cancer deaths as the outcome compared to the models for the common non-cancer causes of death.

Including same week and previous week influenza consultation rates in the model only slightly changed the estimates for temperature and the other environmental variables, although a higher previous week’s influenza rate was a significant predictor of higher mortality rates. Doubling the maximum allowable df for the season term from 4 to 8 did result in a more complex function for season, final df = 6.5 vs. 2.2 for the original model, but the temperature coefficient only increased by 1% and other parameter estimates also changed only slightly. A further sensitivity analysis involved doubling the maximum degrees of freedom for time trend from 4 to 8. This led to a more complex function for long term trend 6.7 df vs 2,9 df for the original model. The estimated mortality increase per 10°C temperature decrease increased slightly to 51% per 10°C decrease, while the estimated ozone effect decreased from 4.0% per 10 μg/m^3^ to 2.4%. Replacing the linear term for average lag 0–20 mean temperature with a smooth term with maximum 4 df slightly decreased the cold wave effect, from 4.0% to 3.2%, while using the original distributed lag term allowing both non-linear effects and flexibility in duration increased the cold wave estimate to 5.5%.

### Taipei

**Figure 2:**
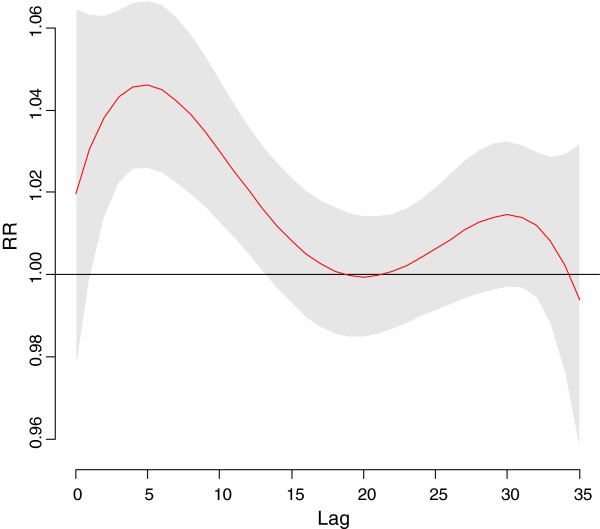
**Relative Risk for mortality by lag in Taipei for a 10°C. day vs, a 25°C. day.** LEGEND: RR = Relative Risk. Relative Risk. 95% Confidence Region for Relative Risks.

The time course of the association between mortality and cold temperatures is shown in Figure 2. Based on the results of the distibuted lag model we then fit a model with a single smooth term representing the average of lags 0–17 mean temperature. Since the association of mortality with this term was approximately linear throughout the range of temperatures for the cool season, the final models used linear terms for average lag 0–17 mean temperature. The results of distributed lag models also indicated that O_3_ and PM_10_ had an effect up to 5 days later so the mean of lags 0–5 was used for these variables. To check for possible residual confounding we also tried the mean of lags 0–17 for these variables, but after confirming that the estimates for the temperature and humidity terms were not affected by this the final model used the mean of lags 0–5 for both pollutants.

The results of these GAMs for mortality in Taipei are shown in Table 3. Lower averages lags 0–17 temperature, higher average lag 0–17 humidity, and higher lag 0–5 O_3_ levels were all significantly associated with higher mortality, while higher lag 0–5 PM_10_ was non-significantly associated with higher mortality (PM_10_ results are not shown in the table as they were non-significant, however PM_10_ was controlled in the models). The results of stratified analyses showed that the effects of temperature and O_3_ were generally stronger for deaths among older age groups, while the cold temperature effect was also stronger for non-cancer deaths. There were no consistent differences between subgroups in RH effects. The model additionally including an indicator for cold wave showed that the days within the cold wave period had an estimated average 3.4% (95% CI = −0.5%, 7.4%, p = .088) mortality after adjustment for all other variables. Thus the estimated effect of a series of days with cold temperatures was non-significantly greater than the expected sum of effects of the individual days. The additional effect of cold waves was higher for males, 4.6%, than for females, 1.9%, and was negligible for the 45–64 and 85+ age groups, but was 2.8% and 9.9% for the 65–74 and 75–84 age groups, respectively. Among common causes of death only deaths due to cardiovascular disease and stroke showed a substantial additional cold wave effect with estimated increases of 15.7% and 8.0% respectively.

**Table 3:** Results of Poisson GAMS without cold wave indicator: Taipei, Taiwan, cool season (January-March and November-December, 1999–2008

	**Lag 0–17 Mean Temperature 10 C drop**		**Lag 0–17 Mean Relative Humidity 10% decrease**		**Lag 0–5 O**_**3**_**10 ppb increase**	
	**% Increase (95)**	**p**	**% Increase**	**p**	**% Increase**	**p**
All	36 (28, 44)	<.001	−2.5(−4.8, -0.1)	.038	3.1 (0.8, 5.5)	.009
Male	37 (26, 48)	<.001	−3.2 (−6.1,-0.1)	.043	3.1 (0.1, 6.1)	.044
Female	33 (21, 45)	<.001	−1.5 (−5.0, 2.1)	.41	3.3 (−0.2, 6.9)	.069
Age						
45-64	13 (2, 25)	.016	1.3 (−3.7, 6.5)	.62	0.5 (−4.0, 5.1)	.83
65-74	28 (14, 44)	<.001	−4.8 (−9.6, 0.0)	.061	3.0 (−1.9,8.2)	.23
75-84	46 (33, 62)	<.001	−2.0(−5.8, 2.0)	.32	2.1 (−1.7, 6.0)	.28
85+	38 (22. 55)	<.001	−2.7 (−7.5, 2.5)	.30	6.4 (1.5, 11.7)	.011
Cause of Death						
Cancer	3 (−7, 14)	.60	−1.7 (−5.7, 2.3)	.45	3.7 (−0.4, 7.9)	.078
Cardiovascular Diseases (excl stroke)	64 (47, 85)	<.001	2.2 (−3.6, 8.3)	.47	3.4 (−1.8, 8.9)	.21
Respiratory	22 (9, 37)	<.001	−2.1 (−7.4, 3.6)	.46	5.5 (0.4, 10.9)	.033
Respiratory infections	80 (44, 124)	<.001	4.0 (−7.2, 16.5)	.49	6.1 (−4.0, 17.3)	.24
Stroke	46 (22, 74)	<.001	−8.9 (−15.9,-2.2)	.011	0.5 (−6.5, 8.1)	.88
Nephritis	42 (13, 79)	.002	−5.6 (−15.9, 5.8)	.32	15.4 (4.3, 27.6)	.005
Septicaemia	21 (−21, 86)	.39	4.0 (−15.6, 28.0)	.71	4.8(−13.4, 26.8)	.63
Diabetes	56 (26, 94)	<.001	−1.8 (−10.2, 7.5)	.67	0.1 (−8.0. 9.0)	.96
Cirrhosis	15 (−14, 56)	.35	0.7 (−14.3,16.7)	.92	−11(−21, 16)	.083

Doubling the maximum df for the seasonality term to 8 reduced the estimated temperature effect only slightly, as did doubling the maximum df for long-term trend. The estimated pollutant effects were also not changed in these models. Use of a smooth term for average lag 0–17 temperature changed the cold spell coefficient by less than 1% while using the distributed lag term increased the estimate from 3.4% to 5.0% but increased the p-value from .088 to .16.

## Discussion

### Comparison between our study cities

Our results concerning cool temperature effects are quite similar for Hong Kong and Taipei, two cities with generally similar cool season climates. While the lagged effects of cool temperatures lasted somewhat longer in Hong Kong, 20 days, than Taipei, 15 days, the estimated cumulative increases in mortality for the two cities were quite close, 45% per 10°C drop for Hong Kong vs. 36% per 10°C drop for Taipei. The subgroup analyses also gave similar results for the two cities, with both showing greater effects for non-cancer causes of deaths and generally greater temperature effects for older age groups, although the mortality increase for the 85+ group was considerably smaller for Taipei than for Hong Kong. The estimated pollutant effects were also similar, with O_3_ being the major pollutant associated with cold season mortality, although the effect size and the duration of lagged effects for Hong Kong were considerably larger. One major difference in the results was the fact that lower RH% was associated with significantly increased mortality in Hong Kong, but significantly decreased mortality in Taipei. This difference is surprising as the distribution of humidity in the cool season is similar between the two cities. A possible partial explanation is that while temperature and humidity are correlated in Hong Kong in the cool season (r = .28) there is no correlation between them in Taipei. Thus the fact that low humidity tends to occur with low temperature in Hong Kong may contribute to its observed association with higher mortality. Differing composition of causes of natural deaths between the two cities may also be a partial explanation. The stratified analyses indicate that the strongest association between higher humidity and higher mortality in Taipei is for stroke deaths. Stroke mortality in Hong Kong was also associated with higher humidity and stroke deaths made up a greater proportion of Taipei natural deaths (10.5%) than Hong Kong deaths (7.1%). Finally random variation may have also played a role in this difference. It should also be noted that the RH associations with mortality in both cities were weak compared to those for the temperature-mortality association.

Comparison of the O_3_ association between Hong Kong and Taipei is complicated by the difference in measurement units as the conversion between ppb and μg/m^3^ depends on air temperature and pressure, but the conversion factor is roughly 1 ppb = 2 μg/m^3^ under average conditions in the cool season in the two cities. Therefore the observed mortality increase of 3.0% per 10 ppb increase in O_3_ observed for Taipei is considerably weaker than the 4.0% mortality increase per 10 μg/m^3^ rises in O_3_ observed for Hong Kong. The mortality increases corresponding to increases in O_3_ from the 25^th^% ile – 75^th^% ile are 3.0% for Taipei and 9.4% for Hong Kong. While sensitivity analyses did show that the estimated ozone effect for Hong Kong was sensitive to the degrees of freedom used for trend variable the conclusion of a strong and significant effect of higher ozone levels on increasing mortality remained unchanged.

### Comparison with other studies

Comparisons with other studies is hampered somewhat by differences in methodology, including differences in meteorological variables considered, statistical analysis methods, lags examined, confounder control, and whether whole-year or season-specific analyses were performed. The ISOTHURM study [4], which looked at temperature effects in several cities in developing countries worldwide, used lags 0–13 mean temperature to evaluate cold weather effects. Their methodology differed from ours in that they consider year round data. One of their studied cities, Monterrey, Mexico, has a sub-tropical climate. The estimated increase in mortality for 1°C drop in lag 0–13 mean temperature was 4.70% below a threshold of 17°C [4], a stronger increase but also a lower threshold than what we observed for Hong Kong and Taipei. Their results for Bangkok, Thailand, which has a tropical climate, showed a somewhat stronger effect size, 4.09% rise in mortality per 1°C temperature decrease, and a high threshold for cold effects, 29°C [4]. Both Hong Kong and Taiwan are more economically developed than either Mexico or Thailand, however it is unclear to what extent this would affect cold weather-mortality associations as many homes in our study locations also do not have central heating. A study from the U.S. included Tampa, a sub-tropical city, and Miami, a tropical city, and the mortality increases per 1°C drop below the ‘minimum mortality temperature’ (MMT) for these cities (around 27°C) were 1.0% and 1.3%, respectively [1]. This is a considerably weaker effect than we observed, however, the authors only considered lags up to 3 days, which may be too little to capture all of the cold effect. A study from Europe [2] used the average of lags 0–15 minimum apparent temperature to estimate cold weather effects on mortality during the cold season, October-March. This study did not include any cities with sub-tropical or tropical climates but did find a stronger effect for the warmer ‘Mediterranean’ cities, 1.62% natural mortality rise per 1°C temperature drop, than for ‘North-Central’ cities, 1.16% [2]. Similar to our study they found that cold weather was more strongly associated with deaths among older people, and for deaths due to respiratory or cardiovascular disease [2]. A study from a sub-tropical area of rural Bangladesh which used the average of lags 0–13 mean temperature estimated a 3.2% increase in natural mortality per 1°C drop in temperature across the temperature range observed, with stronger associations for cardiovascular and respiratory mortality and mortality among children and the elderly [6]. This is quite close to our estimates for Hong Kong and Taipei despite a very different mortality profile for this area, nearly one-third of the deaths occurred among children 0–14 years of age [6].

Many of the previous studies which examined the association between specific cold waves and mortality did not adjust for the effects of individual cold days. Studies from the Netherlands [7], Czech Republic [8] and Russia [9], all found significant excess mortality during cold spells but since the short-term effects of daily temperature were not adjusted it is unsure whether or not some of the excess represented the additional effect of having several cold days consecutively. A study using data from 3 cities in Guangdong province, China examined the short-term effect of a severe 2008 cold wave, which also affected Hong Kong, and found that mortality was increased by 60% in two of the cities during and immediately after the cold wave compared to similar periods for 2006–7 and 2009 [10]. This study also did not adjust for the general effect of cold temperatures. A recently published study [18] using data from 1994–2007 for four Taiwanese cities, including Taipei, found no additional effect of cold waves on mortality once the effects of individual cold temperature days, with lags up to 30 days, were accounted for. A study using data from 1987–2000 for 99 U.S. cities examined the additional effect of cold waves after adjustment for the cold temperature mortality response [19]. This study found no additional cold wave effect and in fact found a small but statistically significant decrease in deaths for the ‘coldest’ cold waves, those with temperatures below the 1^st^ percentile [19]. Our study found an additional cold wave effect on raising mortality after controlling for the general effect of cold temperatures which was significant in Hong Kong and borderline significant in Taipei. Our finding implies that there is evidence that the effect of cold days is greater if they occur in sequence. One possible explanation for the difference between our finding and those from the Taiwanese and U.S. studies is differences in the definition of cold waves. The Taiwanese study, while considering several temperature thresholds and minimum number of consecutive cold days when defining cold waves [18], did not consider the potential lagged effects of cold waves, i.e. the possible persistence of increased mortality after the cold wave has finished. Given that substantial and prolonged lagged effects exist for cold temperatures in general it seems reasonable to assume that they would also exist for cold waves. The U.S. study did consider the possible lagged effects of cold waves, up to 7 days [19], but used a looser definition of cold waves, 2 consecutive days with cold temperature, vs. 5 for our study. In addition our cold wave indicator did not take a value of 1 until the 5^th^ consecutive day of cold temperatures. We feel that this is appropriate since the fact that the first few days of cold weather eventually became part of a cold spell would not retroactively affect mortality on those days. Also most of the cities included in the U.S. study had considerably colder climates than our study cities, and thus would be expected to have weaker cold temperature, and possibly cold wave, effects on mortality.

Ozone levels have been found to have short-term associations with mortality in many studies. A meta-analysis of the results of 43 studies found that most observed a positive association between ozone and mortality [20], although considerable heterogeneity was observed. Their pooled estimate was for a 1.6% increase in natural mortality for each 20 ppb increase in 24-hour mean ozone [20]. Our estimated ozone effects were considerably stronger, especially for Hong Kong.

### Potential scientific explanations for the associations observed in this study

Cold stress has been found to result in blood pressure rises [21-23], cardiac hypertrophy [22], increased blood viscosity [21], and increased platelet counts [21,23]. A prospective study looking at the association between air temperature and risk factors for ischaemic heart disease concluded that the most important effects of cold temperature were on the haemostatic system, including increases in fibrinogen, and α_2_ macroglobulin [23]. A French study found that outdoor temperature and blood pressure were strongly correlated in the elderly [24]. The elderly also have a higher prevalence of co-morbidities which would make them more sensitive to cold effects.

One reason cold days occurring in sequence may have a greater effect than when they occur in isolation may have to with the fact that many homes and workplaces in the study cities do not have central heating. Individual cold days occurring in the midst of several relatively warm days may not lower the temperature of indoor spaces in these cities very much. However several cold days in a row would result in indoor spaces becoming quite cold in the absence of a heating source. Thus the actual exposure to cold temperatures could be greatly increased by having the cold days occur in succession. This would be particularly true for subpopulations that are not particularly active, such as the elderly or those with chronic diseases.

Our findings regarding the association between relative humidity and mortality were inconsistent across cities and between different causes of death. Lower relative humidity has been found to be more favorable for transmission of the influenza virus [25] and this could lead to increases in mortality. The observed protective effect of higher solar radiation in reducing cold season mortality in Hong Kong may reflect the potential effects of sunshine to warm buildings and outdoor urban areas.

The observed ozone-mortality associations for our study were especially strong for respiratory and cardiovascular mortality, particularly for Hong Kong. There is evidence that exposure to ozone causes decreases in lung function due to reductions in inspiration capacity caused by sensitization of bronchial C-fibers and small airway narrowing [26]. A recent controlled-human-exposure study [27] found that ozone exposure was associated with increases in vascular inflammation markers and pulmonary inflammation, decreases in lung function and changes in markers of fibrinolysis and markers that affect autonomic heart rate control.

Our study results indicate that cold temperatures result in substantially elevated short-term mortality rates in these sub-tropical Asian cities. Although cold weather cannot be prevented steps can be taken to reduce the exposure of vulnerable groups to cold temperatures. Promoting further awareness of the adverse health effects of cold weather among the public could help persuade individuals to take steps to reduce their exposure, including dressing more warmly, and keeping their homes and offices warmer. Measures to reduce excess mortality and morbidity have been found to be effective for hot weather [28-31] and should be for cold weather, e.g. issuing cold weather warnings, provision of warm public places and suitable clothing, particularly for people at high risk such as the elderly and those with chronic diseases. Since temperatures can be forecast a few days in advance a warning system could be set in which people belonging to high risk groups could be warned in advance when weather conditions with the potential to cause adverse health effects are coming [31].

## Conclusions

Our study results indicate that cold temperatures results in substantially elevated short-term mortality rates in Hong Kong and Taipei which have sub-tropical climates with a great deal of temperature variability in the winter. Although the winter temperatures experienced in these cities are relatively mild in comparison to those observed in Northeast Asia, Europe or North America, the strength of the association between temperature drops and mortality is generally much stronger, presumably due to the lesser extent of acclimatization, both environmental and physiological, for populations living in warmer areas. We also found that cold waves had an independent association with higher mortality after controlling for the effects of individual cold days, indicating that cold days in sequence had a stronger effect on mortality.

## Abbreviations

DOY: Day of the year; DOW: Day of the week; GAM: Generalized additive Model; RH: Relative humidity.

## Competing interests

The authors declare that they have no competing interests.

## Authors’ contributions

WG conceived the study, participated in the statistical analysis and the drafting of the manuscript. CY and EC contributed to the acquisition of the data, design of the study, drafting and critical revision of the manuscript. MC participated in the statistical analysis and drafting and critical revision of the manuscript. All authors read and approved the final manuscript.
